# Association Between Vitamin D Levels and Developmental Status in Short-Stature Children

**DOI:** 10.3390/children11121542

**Published:** 2024-12-19

**Authors:** Mia Milanti Dewi, Akhmad Imron, Nelly Amalia Risan, Grace Mediana Purnami, Raden Tina Dewi Judistiani, Budi Setiabudiawan

**Affiliations:** 1 Departement of Child Health, Faculty of Medicine, Universitas Padjadjaran, Bandung 40161, Indonesia; nelly.amalia@unpad.ac.id (N.A.R.); budi.setiabudiawan@unpad.ac.id (B.S.); 2 Department of Neurosurgery, Faculty of Medicine, Universitas Padjadjaran, Bandung 40161, Indonesia; a.imron@unpad.ac.id; 3 Study Center of Immunology, Faculty of Medicine, Universitas Padjadjaran, Bandung 40161, Indonesia; gracemediana1412@gmail.com; 4 Department of Public Health, Faculty of Medicine, Universitas Padjadjaran, Bandung 40161, Indonesia; tina.d.judistiani@unpad.ac.id; 5 Faculty of Medicine, President University, Bekasi 17550, Indonesia

**Keywords:** children, development, short stature, vitamin D

## Abstract

**Background:** The first two years of life are crucial for child growth and development, and short stature is a common issue influenced by nutritional deficiencies. This study aimed to determine the relationship between vitamin D levels and short stature in children under 2 years of age in Bandung. **Methods:** Conducted as part of the ALG Grant research entitled ‘*The Role of Vitamin D in Reducing Maternal and Infant Mortality Rates Across Bandung Regency*’, this case-control study included 221 children, of whom 46.1% were below average height. **Results:** Nutritional status played a significant role, with short stature being associated with underweight (21.6%), severely underweight (29.4%), wasted (12.7%), and severely wasted (10.8%) conditions. However, the analysis revealed no significant difference in vitamin D levels between children with short stature and those with normal stature (median 28.8 vs. 28.8, *p* = 0.555). Logistic regression showed that short stature increased the risk of developmental deviations by 5.46 times compared to normal stature. **Conclusions:** While short stature remains a concern in Bandung, vitamin D levels do not appear to influence the incidence of short stature or developmental deviations in this population.

## 1. Introductions

According to the World Health Organization (WHO) Child Growth Standards (CGS), short stature is identified when children are −2 standard deviations (SDs) or more below the height for their chronological age or less than the third percentile [1]. Stunting, on the other hand, is defined as a condition resulting from chronic malnutrition that leads to impaired linear growth and development, typically measured in children under the age of 5 years [2]. Although both terms involve height, stunting emphasizes the nutritional and developmental context, while short stature is more general and includes other possible causes such as genetic or hormonal factors. Globally, approximately 162 million children under the age of 5 have short stature [2]. In Indonesia, this prevalence in 2022 was 21.6% based on data from the Indonesian Nutrition Status Survey (SSGI), with an incidence of 25.0% in the Bandung district, ranking fifth in West Java [3]. In working towards achieving the goals of sustainable development, the Indonesian government is working to reduce these figures [2,4,5,6,7].

The short stature that occurs in the first 1000 days of life is caused by various factors, including micronutrient deficiencies such as vitamin D [1,4]. While micronutrient deficiencies such as vitamin D have gained attention as contributors, other factors—such as chronic infections, maternal malnutrition, low birth weight, and suboptimal feeding practices—also play significant roles in impairing linear growth [1,4,5,6]. Socio-economic disparities further compound these issues, limiting access to adequate healthcare and nutrition.

Vitamin D is a fat-soluble micronutrient that functions in the intestinal absorption of calcium and is believed to play a role in the growth of children. Vitamin D deficiency can have short-term and long-term effects. Vitamin D can affect the nervous system, and it has a role in the skeletal system. Vitamin D plays a role in brain health from the womb to old age [7]. Vitamin D receptors and 1α hydroxylase are located in neuronal and glial cells, in the nucleus and cytoplasm, and in the cingulate gyrus, caudate, putamen, and substantia nigra [8]. In vitro studies have shown that vitamin D has various actions on oligodendrocytes, astrocytes, microglia, and neuronal cells [8]. Vitamin D-deficient pregnancies can lead to premature birth, low birth weight, and hypoxic brain injury [9].

The prevalence of vitamin D deficiency in Asia is approximately 70%, varying between 6 and 70% in Southeast Asia, >50% in Indonesia, and 47–75% in Malaysia [7,8,9,10]. Children (neonates, preschool age) are often more at risk of vitamin D deficiency than adults [9,10,11,12]. The clinical manifestations of vitamin D deficiency are highly variable and even unknown [13,14,15,16,17,18,19,20].

Despite the potential link between vitamin D deficiency and short stature, findings across studies remain inconsistent. While research in Ecuador identified an association between low vitamin D levels and short stature in children aged 6–36 months, studies in Bangladesh found no significant differences in vitamin D levels among children with and without short stature [21,22,23,24]. These discrepancies underscore the need for further exploration of the multifactorial etiology of short stature and the role of vitamin D in developmental deviations. Additionally, delays in developmental milestones, frequently seen in vitamin D-deficient children, may serve as early warning signs that require timely intervention.

## 2. Materials and Methods

This prospective cross-sectional study was conducted to evaluate the role of vitamin D in the developmental delays of children under two years of age with a history of stunted growth in Bandung Regency, West Java, Indonesia. The study targeted participants from the ALG research grant titled “*The Role of Vitamin D in Reducing Maternal and Infant Mortality Rates Across Bandung Regency*”. Children under 2 years of age with a height-for-age z-score (HAZ) < −2SD based on the WHO CGS curve were included. Exclusion criteria included abnormal delivery processes, prematurity, low birth weight, history of seizures, multiple congenital anomalies, syndromic conditions, or chronic diseases and congenital anomalies that could affect growth and development, such as congenital heart defects and neurological disorders. Developmental status was assessed using the Indonesian standardized Prescreen Development Questionnaire (PSDQ), categorizing results as normal or abnormal. Ethical approval was obtained from the Research Ethics Committee of the Faculty of Medicine, Universitas Padjadjaran.

Serum samples were collected and analyzed to measure vitamin D levels using enzyme-linked immunosorbent assay (ELISA) kits. The stored biological samples, initially collected during the ALG study, were maintained at −80 °C. Vitamin D levels were categorized as deficient (<20 ng/mL), insufficient (21–29 ng/mL), or sufficient (>30 ng/mL). Developmental assessments were conducted in conjunction with laboratory analyses performed at the Clinical Pathology Laboratory of Dr. Hasan Sadikin General Hospital, Bandung.

The measurement of 25-hydroxyvitamin D [25(OH)D] was performed using the Euroimmun ELISA kit (Euroimmun AG, Lübeck, Germany). Serum samples were processed according to the manufacturer’s protocol, and absorbance was read at 405 nm using an ELISA reader (BioTek Instruments, Winooski, VT, USA). Sample size calculations were based on a 95% confidence level and 80% statistical power, resulting in a required minimum of 30 participants per group.

Before conducting statistical analyses, the normality of the data was assessed using the Shapiro–Wilk test to determine the appropriateness of parametric or non-parametric tests. Based on the results, the data in this study were not normally distributed. Descriptive statistics were used to summarize nutritional status and the prevalence of short stature. Comparisons of vitamin D levels between groups were made using the independent t-test for normally distributed data or the Mann–Whitney U-test for non-normally distributed data. Logistic regression was employed to assess the association between short stature and developmental deviations, with results expressed as odds ratios (ORs). A *p*-value of <0.05 was considered statistically significant. All analyses were performed using SPSS version 24.

## 3. Results

A total of 221 children, consisting of 102 (46.2%) with short stature and 119 (53.8%) with normal stature, participated in this study. The characteristics of the subjects are shown in Table 1.

The median age of the short-stature group was significantly higher than that of the normal-stature group (19 months vs. 14 months, *p* < 0.001). The sex distribution in both groups was predominantly male. Mothers of short-stature children mainly had an education level of elementary or junior high school, while those of normal-stature children predominantly had a high school education or higher. Short-stature children were classified as underweight (21.6%), severely underweight (29.4%), or normal weight (49.0%), compared to normal-stature children, who were predominantly normal weight (91.6%), with only 7.6% underweight and 0.8% severely underweight. Based on height-to-weight ratio, 12.7% and 10.8% of short-stature children were wasted and severely wasted, respectively, while 76.5% had normal ratios. In contrast, 87.4% of normal-stature children had normal ratios, with 10.1% wasted and 2.5% severely wasted. Head circumference was similar across groups.

Table 2 shows no significant differences in vitamin D levels (median 28.8 vs. 28.8, *p* = 0.555) or median age (17 vs. 12 months, *p* = 0.067) between short- and normal-stature children. Nutritional status based on BB/U of children with PSDQ deviation in short children included 15.8% underweight, 15.8% severely underweight, and 68.4% normal, compared to 93.5% normal in children of normal stature, with only 3.2% underweight and severely underweight. Height-for-age ratios showed 22.2% stunted, 29.9% severely stunted, and 48.9% normal among short children, while normal PSDQ children had 83.9% normal, 12.9% stunted, and 3.2% severely stunted. PSDQ screening gave body weight and head circumference similar to age.

Table 3 shows higher deviations in children with short stature than in children with normal stature (95.1% vs. 78.2%, *p* < 0.001). However, there was no difference in vitamin D levels between children with different PSDQ scores (median 28.8 vs. 29.9, *p* = 0.4313). Likewise, there was no difference in the proportion of children who experienced vitamin D deficiency, insufficiency, or normal levels (86.7% vs. 86.2% vs. 85.6%, *p* = 0.985).

The logistic regression analysis in Table 4 showed that children with short stature have an increased risk of deviations of 5.46 times compared to those with normal stature. However, vitamin D had no effect on the incidence of PSDQ deviations.

## 4. Discussion

The study identified 102/221 (46.1%) children of short stature with mothers with lower education levels than those of children of normal height. This is similar to a study that revealed that maternal education was correlated with children of short stature in Indonesia [6]. Thus, more effort is needed from the government to improve education for mothers.

Anthropometric measures of short children revealed some underweight, severely underweight, and stunted children. Macronutrient and micronutrient deficiencies leading to lower body weight may also negatively impact height. A study in Malaysia found that 78.6% of children under 5 years of age were underweight. This can be influenced by lack of parental education, maternal nutrition, number of children, poverty, access to health facilities, and differences in conditions between cities and districts [25,26,27,28,29,30].

Vitamin D is a fat-soluble micronutrient that functions in the intestinal absorption of calcium and is believed to play a role in the growth of children. Vitamin D deficiency has been widely reported worldwide; however, the trends are inconsistent. Research in Bangladesh did not show differences in vitamin D levels in children with or without short stature [21]. However, vitamin D was associated with short stature in children aged 6 to 36 months in Ecuador [22]. Meanwhile, in India, vitamin D supplementation in children with short strides in India increased child growth [22,23].

In our study, there were no differences in vitamin D levels in both groups. However, vitamin D deficiency and insufficiency were found. Although Indonesia has sun exposure that provides sufficient vitamin D, some children experience vitamin D deficiency. Vitamin D exposure must be increased through outdoor play between 10:00 and 13:00 without the use of head covers or sunscreen and allowing more limbs to be exposed to sunlight.

Vitamin D deficiency can have short-term and long-term effects. Vitamin D can affect the nervous system and its role in the skeletal system. Vitamin D plays a role in brain health from the womb to old age [31]. Vitamin D receptors and 1α hydroxylase are located in neuronal and glial cells, in the nucleus and cytoplasm, and in the cingulate gyrus, caudate, putamen, and substantia nigra [30]. In vitro studies have shown that vitamin D has various actions on oligodendrocytes, astrocytes, microglia, and neuronal cells [32]. Vitamin D-deficient pregnancies can lead to premature birth, low birth weight, and hypoxic brain injury [32].

The biological pathways of vitamin D demonstrate several functions in the central nervous system, such as increasing brain cell differentiation, neurotrophin expression, neurotransmitter synthesis, intracellular calcium signaling, and gene/protein expression related to neuron structure. Research in rats born with vitamin D deficiency has shown anatomical changes in the brain, with an expanded cortex area (30%) and decreased ventricular volume, indicating a distortion in early brain development [31].

According to the recommendations of the American Academy of Pediatrics (AAP), developmental screening should be performed regularly, especially at the ages of 9, 18, and 30 months [33,34]. The PSDQ developed in Indonesia has good sensitivity and specificity and was therefore used to link short stature and vitamin D levels.

This study showed that developmental deviations were more prevalent among short children (95.1% vs. 78.2%, *p* < 0.001). There was no difference in vitamin D in the development deviation group. The relationship between vitamin D and developmental deviations has been studied, but the results are inconsistent. A delay in developmental milestones may be one of the early symptoms that accompany vitamin D deficiency [35]. Fetal vitamin D can affect brain development, as determined by the use of ASQ at 6 months, 12 months, and 24 months of age [26]. Vitamin D administration is also positively associated with temperament, language, and behavior but negatively associated with cognitive and motor impairment [21]. Vitamin D deficiency during the first trimester increases the risk of motor delays by 1 year of age [27]. On the other hand, growth or development in children with vitamin D deficiency has not shown clear differences [28]. In this study, logistic regression analysis showed that short children have a 5.46 times higher risk of developmental deviations.

## 5. Conclusions

Short stature remains a problem in the Bandung district of Indonesia. Although vitamin D levels do not affect height and developmental deviations, short children are more likely to be underweight and wasted, with a higher incidence of developmental deviations. It should be noted that one of the limitations of this study is that it did not analyze other factors affecting growth or children experiencing developmental deviations such as parenting, diet, genetic factors, and other nutritional elements such as calcium and phosphorus. Therefore, further research is warranted on the relationship between vitamin D and short stature and developmental deviations.

## Figures and Tables

**Table 1:** Characteristics of short and normal stature children.

Variable	Short Stature *n* = 102	Normal Stature *n* = 119	*p*
**Age (Month)**			
Median (IQR)	19 (15–22)	14 (9–20)	<0.001 ^a^*
**Sex**			
Male	61 (59.8)	63 (52.9)	0.305 ^b^
Female	41 (40.2)	56 (47.1)	
**Mother’s Education**			
Elementary	41 (40.2)	31 (26.1)	0.010 ^b^*
Junior high school	43 (42.2)	47 (39.5)	
Senior high school	18 (17.6)	41 (34.5)	
**Mother’s Occupation**			
Housewife	90 (88.2)	111 (93.3)	0.193 ^b^
Working	12 (11.8)	8 (6.7)	
**Socio-economic status**			
Minimum wage	5 (4.9)	5 (4.2)	0.590 ^b^
Below	87 (85.3)	97 (81.5)	
Above	10 (9.8)	17 (14.3)	
**Body weight/age**			
Normal	50 (49.0)	109 (91.6)	<0.001 ^b^*
Underweight	22 (21.6)	9 (7.6)	
Severely underweight	30 (29.4)	1 (0.8)	
**Nutritional Status (weight/height)**			
Normal	78 (76.5)	104 (87.4)	0.029 ^b^*
Wasted	13 (12.7)	12 (10.1)	
Severely wasted	11 (10.8)	3 (2.5)	
**Head Circumference**			
Normal	75 (73.5)	84 (70.6)	0.200 ^b^
Microcephaly	23 (22.5)	23 (19.3)	
Macrocephaly	4 (3.9)	12 (10.1)	

Note: ^a^ Mann–Whitney test, ^b^ Chi-square test, * significant difference.

**Table 2:** Differences in vitamin D levels between children of short stature and normal height.

Variable	Short Stature n = 102	Normal Stature n = 119	*p*
**Vitamin D**			
Median (IQR)	28.8 (23.6–32.5)	28.8 (22.8–36.2)	0.555 ^a^
**Vitamin D**			
Deficiency	12 (11.8)	18 (15.1)	0.647 ^b^
Insufficiency	43 (42.1)	44 (37.0)	
Normal	47 (46.1)	57 (47.9)	

Note: ^a^ Mann–Whitney test, ^b^ Chi-square test.

**Table 3:** Characteristics of Prescreen Development Questionnaire (PSDQ) for child development.

Variable	PSDQ		*p*
	Deviation/Doubtful *n* = 190	Normal *n* = 30	
**Age (month)**			
Median (IQR)	17 (12–21)	12 (9–20)	0.067 ^a^
**Sex**			
Male	106 (55.8)	18 (58.1)	0.813 ^b^
Female	84 (44.2)	13 (41.9)	
**Mother’s Education**			
Elementary school	65 (34.2)	7 (22.6)	0.213 ^b^
Junior high school	78 (41.1)	12 (38.7)	
Senior high school	47 (24.7)	12 (38.7)	
**Mother’s Occupation**			
Housewife	171 (90.0)	30 (96.8)	0.322 ^b^
Working	19 (10.0)	1 (3.2)	
**Socio-economic status**			
According to regional minimum wage	9 (4.8)	1 (3.2)	0.160 ^b^
Below regional minimum wage	161 (84.7)	23 (74.2)	
Above regional minimum wage	20 (10.5)	7 (22.6)	
**Body Weight/Age**			
Normal	130 (68.4)	29 (93.5)	0.015 ^b^*
Underweight	30 (15.8)	1 (3.2)	
Severely underweight	30 (15.8)	1 (3.2)	
**Height/Age**			
Normal	93 (48.9)	26 (83.9)	0.001 ^b^*
Stunted	42 (22.2)	4 (12.9)	
Severely stunted	55 (29.9)	1 (3.2)	
**Nutrition State BB/TB**			
Normal	153 (80.6)	29 (93.6)	0.204 ^b^
Wasted	24 (12.6)	1 (3.2)	
Severely wasted	13 (6.8)	1 (3.2)	
**Head Circumference/Age**			
Normal	135 (71.1)	24 (77.4)	0.610 ^b^
Microsefal	40 (21.0)	6 (19.4)	
Macrosefal	15 (7.9)	1 (3.2)	

Note: ^a^ Mann–Whitney test, ^b^ Chi-square test, * significant difference.

**Table 4:** Relationship between short stature, vitamin D, and child development according to logistic regression analysis.

Variable	Total	PSDQ		*p*	Univariate		Multivariate	
	*n* = 221	Deviation/Doubtful	Normal		OR (95% CI)	Nilai *p*	Adjusted OR (95% CI)	*p*
		*n* = 190	*n* = 31					
**Stature**								
Short	102	97 (95.1)	5 (4.9)	<0.001 ^b^*	5.42 (2.00–14.72)	0.001 *	5.46 (2.01–14.84)	0.001 *
Normal	119	93 (78.2)	26 (21.8)		1		1	
**Vitamin D**								
Median (IQR)		28.8 (22.975–34.6)	29.9 (25.3–35.3)	0.413 ^a^				
**Vitamin D**								
Deficiency	30	26 (86.7)	4 (13.3)	0.985 ^b^	1.10 (0.34–3.59)	0.88	1.19 (0.35–4.01)	0.784
Insufficiency	87	75 (86.2)	12 (13.8)		1.05 (0.46–2.39)	0.901	0.99 (0.43–2.31)	0.983
Normal	104	89 (85.6)	15 (14.4)		1		1	

Note: ^a^ Mann–Whitney test, ^b^ Chi-square test, * significant difference. Dependent variable: Development deviation, OR = odds ratio, CI = confidence interval.

## Data Availability

The original data used to support the findings of this study are available upon request from the corresponding author due to ethical reasons.
